# An exclusive in-situ dataset on physicochemical parameters in the gappy northern Bay of Bengal

**DOI:** 10.1016/j.dib.2020.106024

**Published:** 2020-07-14

**Authors:** Md Masud-Ul-Alam, Ashif Imam Khan, Saif Khan Sunny, Atiqur Rahman, Muhammad Shahinur Rahman, Bayzid Mahmud, Ashifur Rahman Shaheen

**Affiliations:** a Department of Oceanography and Hydrography, Bangabandhu Sheikh Mujibur Rahman Maritime University, Plot# 14/06-14/23, Pallabi, Mirpur-12, Dhaka 1216, Bangladesh; b Physical and Space Oceanography Department, Bangladesh Oceanographic Research Institute, Marine Drive Road, Jhaliapalong Mouza, Ramu, Cox's Bazar 4700, Bangladesh

**Keywords:** Temperature, salinity, Density, Mixed layer depth, Northern Bay of Bengal

## Abstract

In-situ measurement of physical characteristics of seawater (temperature, salinity, and density) collected with a single fire module CTD (Conductivity, Temperature, Depth) in the northern Bay of Bengal (NBoB), as a part of the Indian Ocean, are given in this article. Due to the scanty of data in the NBoB, such in-situ measurements will certainly play a crucial role in understanding regional oceanic processes. The presented data were collected onboard during two cruises covering 12 stations with high resolution (0.2 m depth) up to 50-meter contour depth from 20.5778 N, 92.3578 E to 21.287 N, 89.696 E. We present raw CTD data as ASCII (.dat) and Comma Separated Value (.csv), and processed CTD data as netCDF (.nc) format in this article. Additionally, the calculated Mixed Layer Depth that was derived from the netCDF (.nc) file was included in the tabular format. This processed in-situ data would be most useful as a baseline for further studies in coastal and offshore physicochemical properties in the NBoB.

**Specifications Table**

**Table:** 

Subject	Earth and Planetary Sciences: Oceanography
Specific subject area	Physical, and acoustical oceanography; model validation; coastal process
Type of data	Tables, Figures, Graphs, Files
How data were acquired	In-Situ Measurement of temperature, salinity, density Instruments: i) Single fire module with CTD, ii) Memory probe Model and make of the instruments used: i) Sea & Sun Technology GmbH ii) CTD90M HYDRO-BIOS Apparatebau GmbH, Germany. Software: Standard data acquisition software SST-SDA Sea & Sun Technology GmbH, Germany.
Data format	Raw, Analyzed, Filtered
Parameters for data collection	Stations positions (latitude and longitude), Conductivity, Temperature, Depth, Salinity, and Density
Description of data collection	These in-situ data were obtained on-board Bangladesh Navy Ship Sangu and fishing vessel Salman-2 by taking CTD profiles at pre-determined 12 stations, ranging throughout the northern tip of the Bay of Bengal.
Data source location	Region: Northern Bay of Bengal; Country: Bangladesh; Latitude and longitude (and GPS coordinates, if possible) for collected samples/data: (20.5778 N, 92.3578 E to 21.287 N, 89.696 E)
Data accessibility	With the article

**Value of the Data**

• In-situ measurements with the CTD are rare in the northern tip of the Bay of Bengal (BoB). This invaluable dataset of temperature, salinity, and density with corresponding depth can be used as the fundamental data in winter and spring season in the northern bay.
• This data can widely be used in any oceanographic research like mixed layer depth (MLD), thermocline, thermal inversion, stratification, barrier layer in the northern BoB.
• The temperature, salinity, and density profiles will be exclusively used for the validation of satellite data, and the ocean and atmospheric model in this region of highly sedimentation rate and freshwater discharge.
• Profiles were taken comprehensively, covering in such a way that could represent the Bangladesh EEZ for further research.

## 1 Data Description

**Fig. 1:**
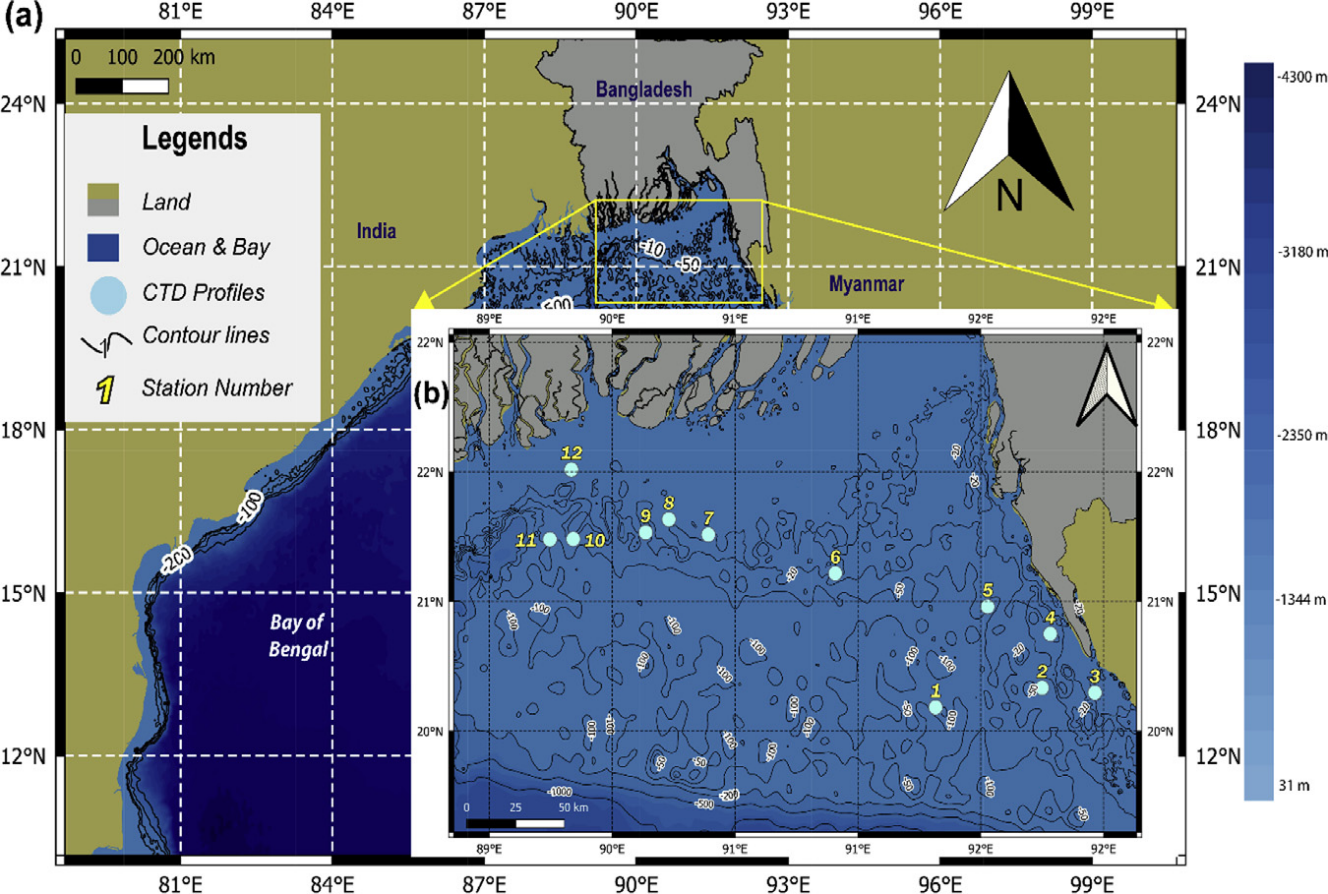
Spatial map of the study area; (a) Bay of Bengal located at the northern tip of the Indian ocean, where darker blue color represents deeper sea while shallower sea is depicted by lighter blue color. Bathymetry was produced using GEBCO Compilation Group (2020) GEBCO 2020 Grid. (b) A closer view to the northern Bay of Bengal illustrated 12 stations numbered with light-blue circles, at which the CTD (Conductivity, Temperature, Depth) profiles were taken onboard.

**Fig. 2:**
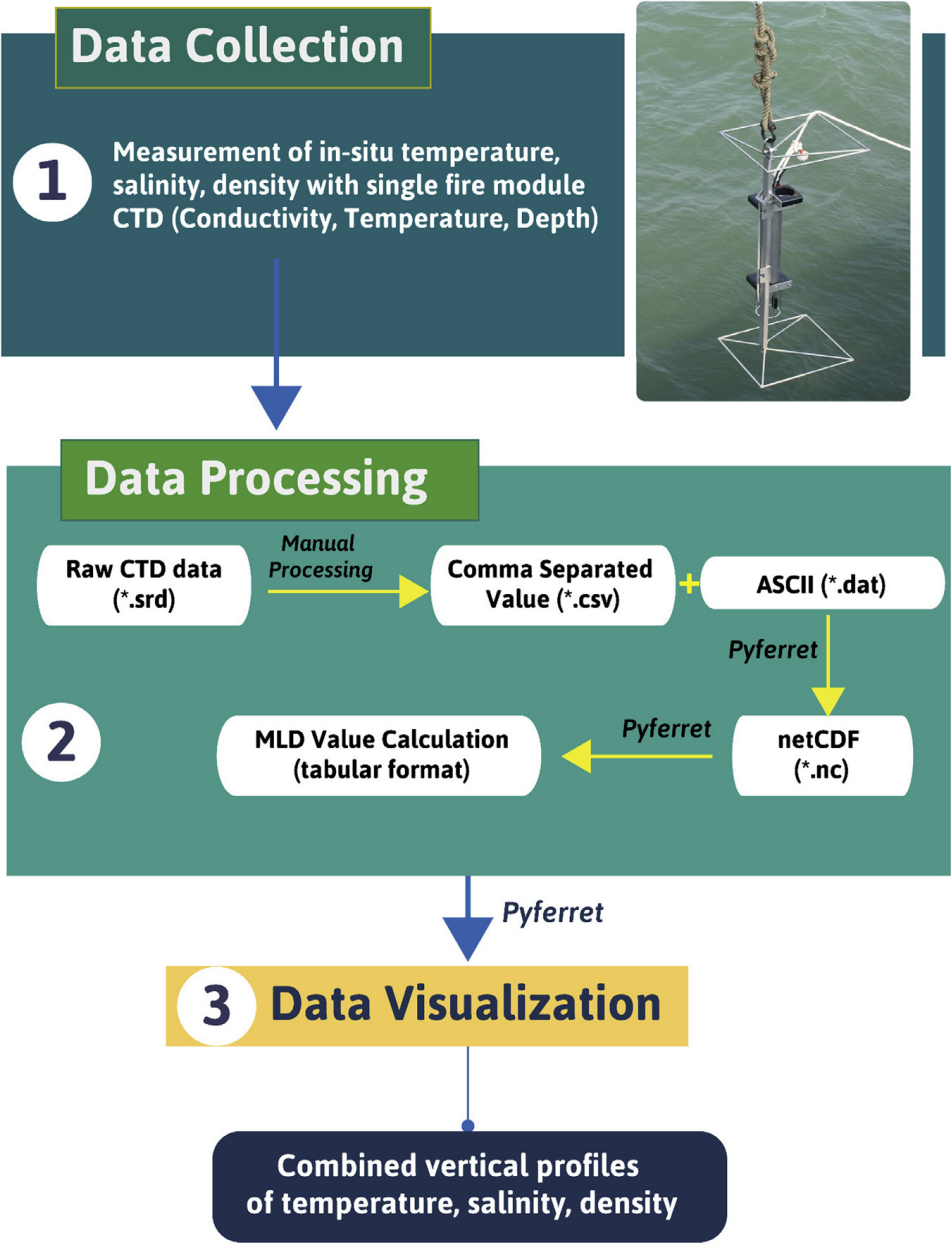
Graphical summary of the three major procedure: data collection, data analysis, data processing; steps to produce the in-situ temperature, salinity, density and Mixed Layer Depth (MLD) values.

The northern tip of the Indian Ocean (Fig. 1(a)), the Bay of Bengal especially its northernmost part, is under studied because of the lack of collaborative research and research vessel which resulting in the paucity and unavailability of in-situ data. Our article addresses that data gap through this dataset, as it predominantly consists of the vertical profile of temperature ( °C), salinity in Practical Salinity Unit (PSU), density (kg m^−3^), and pressure in decibar (dbar) in the predefined 12 stations throughout northern Bay of Bengal (NBoB) (Fig. 1(b)). These oceanographic profiles were collected using CTD which directly measures temperature (accuracy: ± 0.002 °C), conductivity (accuracy: ± 0.002 mS/cm), and pressure (accuracy: up to 0.05% full scale in the range of –5 to 35 °C). At the same time, salinity was derived from conductivity, and density was computed from temperature and salinity [1]. The depth was obtained from the pressure (1dbar equivalent to 1 m depth). The single fire module with CTD (Sea and Sun Technology GmbH) was made and assembled in Germany was employed to collect the data. Initially, data was directly gathered as Sage Report Data File (.srd) format in the built-in memory in CTD. After processing this was transformed into comma-separated values (.csv), text-based ASCII (.dat) file, and eventually in NetCDF (.nc) formats (Fig. 2). In addition to directly measured physical properties of water columns, Mixed Layer Depth (MLD) values were calculated from the derived NetCDF (.nc) files of each station. Stronger vertical stratification and mixing associated with temperature, salinity, especially in the northern BoB, leads to the formation of a shallow mixed layer [2]. Therefore, derived MLD values from in-situ profiles would portray seawater properties of the specified water column more concisely.

In-situ CTD data went through both pre-processing and post-processing stages. The dataset presented here in tabular, digital formats that include:

■ Raw vertical profile data of the water column, which was collected directly by CTD, is presented in Comma-separated value (.csv) files.
■ Manually processed profile values of the parameters of each station are shown in 12 ASCII (.dat) files.
■ Analyzed data of physical parameters' profiles are finally embedded in 12 netCDF files.
■ Mean and Standard Deviation of temperature, salinity, density, and MLD values of the water column are given in tabular format (Table 2).

## 2 Experimental designs, materials, and methods

### 2.1 Data collection with CTD

This data collection was done as an additional task during the cruises for a research project funded by the University Grant Commission, Bangladesh. The cruises took place in the NBoB (Fig. 1(b)) during February and March in 2020 and consisted of a total of 12 stations (Table 1) for the CTD profiles. It was divided into three sessions. First session's profile was taken during a field trip on 02 January 2020. While, second being in 05 to 09 February 2020 which was conducted onboard "Bangladesh Navy Ship Sangu" (BNS Sangu) while the other spanned from 17 to 22 March in the same year, with the help of a fishing vessel named "Salman-2″. Although each session included different vessels, profiles onboard both ships were done using newly procured CTD. This additional memory probe is a medium-sized microprocessor-controlled multi-parameter probe for precise measurements up to 2000 m water depth as well as containing a 16-channel data acquisition system with 16-bit resolution. A flash memory card of 128 megabytes’ capacity stores data.

**Table 1:** Environmental conditions and the geographical coordinates of the 12 stations.

Station no.	Date	Latitude	Longitude	Time	Depth (m)	Wind direction	Current direction	Tide	Wind speed (ms^−1^)
1	18/03/20	20.51 N	90.58 E	12:31 p.m.	50.096	090	085	2nd hrs of ebbing	09
2	08/02/20	20.6 N	92.1 E	09:38 a.m.	22.956	350	155	2nd hrs of ebbing	23
3	02/01/20	20.5778	92.3578	5:17 p.m.	15.16	300	290	5th hrs of flooding	10
4	19/03/20	20.85 N	92.14 E	11:53 a.m.	48.865	030	155	3rd hrs of ebbing	24
5	19/03/20	20.976 N	91.834 E	06:07 p.m.	43.833	375	115	5th hrs of ebbing	24
6	21/03/20	21.13 N	91.09 E	10:45 a.m.	27.953	345	340	Last hrs of flooding	25
7	09/02/20	21.309 N	90.47 E	11:06 a.m.	48.686	330	215	3rd hrs of ebbing	17
8	09/02/20	21.38 N	90.277 E	08:54 a.m.	50.506	030	035	Last hrs of flooding	22
9	09/02/20	21.319 N	90.164 E	09:56 a.m.	32.823	330	215	1st hrs of ebbing	20
10	09/02/20	21.2884 N	89.8102 E	11:57 a.m.	45.653	040	215	5th hrs of flooding	10
11	09/02/20	21.287 N	89.696 E	04:42 p.m.	21.135	330	215	3rd hrs of flooding	05
12	09/02/20	21.61 N	89.801 E	03:50 p.m.	27.809	035	215	2nd hrs of flooding	04

All precautionary steps were followed according to the manufacturer's guidelines to prepare the sensors from atmospheric to aquatic environments to get data properly. Before each casting, the settings of CTD was checked and programmed with a laptop onboard so that measurements remained more precise. The program was set in such a way that for getting five values of each meter of depth (every 0.2 dbar increasing pressure will record one value). It requires some time for the probe to start taking readings even after it's lowered down to the water; therefore, first few readings might get erroneous (i.e., first one meter in the surface water). Therefore, the CTD sensors were acclimatized into the surface of the water for a period of 10 s to get the sensors work properly. A summary of the physical characteristics water column in each station is given in Table 1.

### 2.2 Data processing

In-situ data was obtained via CTD which was stored in the memory probe's flash drive as Sage Report Data File (.srd) format; each CTD deployment generated a (.srd) file with supplementary metadata. Standard data acquisition software SST-SDA was used onboard laptop, which records, displays, and maneuvers collected data. With the help of this software, binary data (.srd) were gathered from a flash drive and were processed and converted into a more accessible format, Comma Separated Value (.csv). While deploying CTD from the vessel, it has to pass through two entirely different environments (from atmosphere to ocean). Therefore, the sensors of the device required some time (10–20 s) for adjusting to the oceanic condition. During this period, it also took erroneous readings. In later steps, these erroneous values, especially the first few readings while deployment and other unusual values (e.g. negative values), were manually omitted, and values starting from 1-meter pressure level were considered for further processing. Furthermore, a few profiles were taken as an average of every one-meter depth values.

**Fig. 3:**
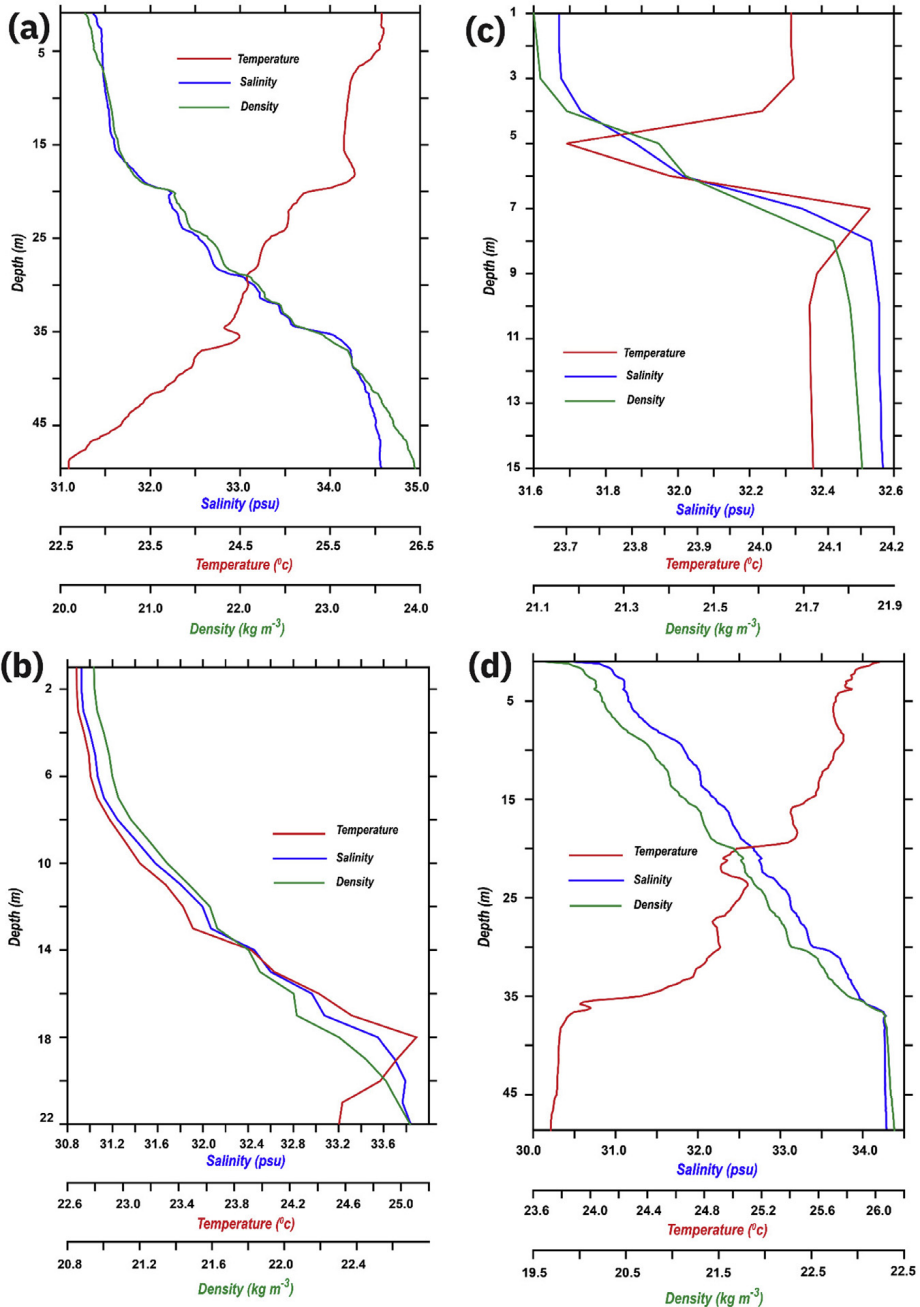

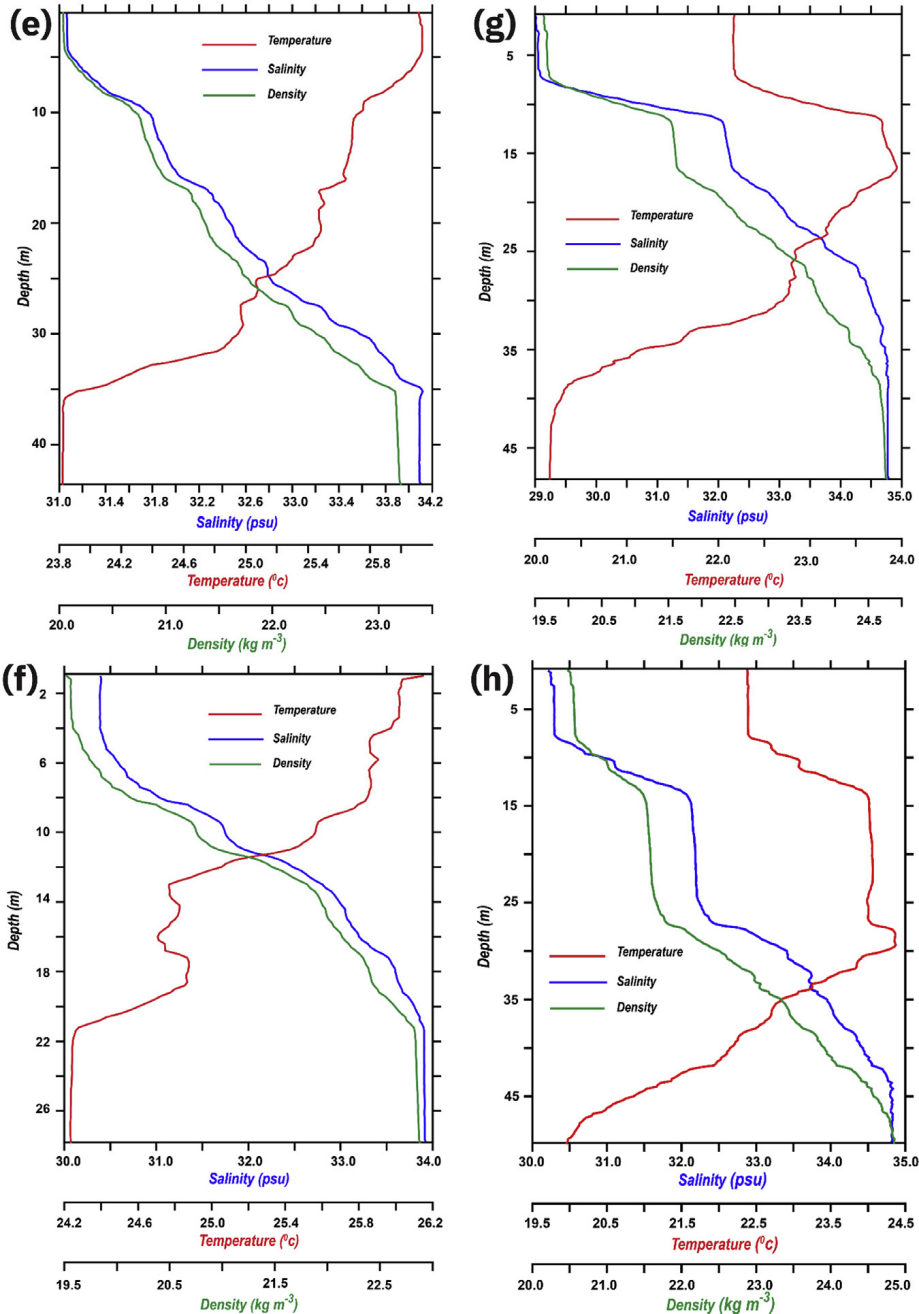

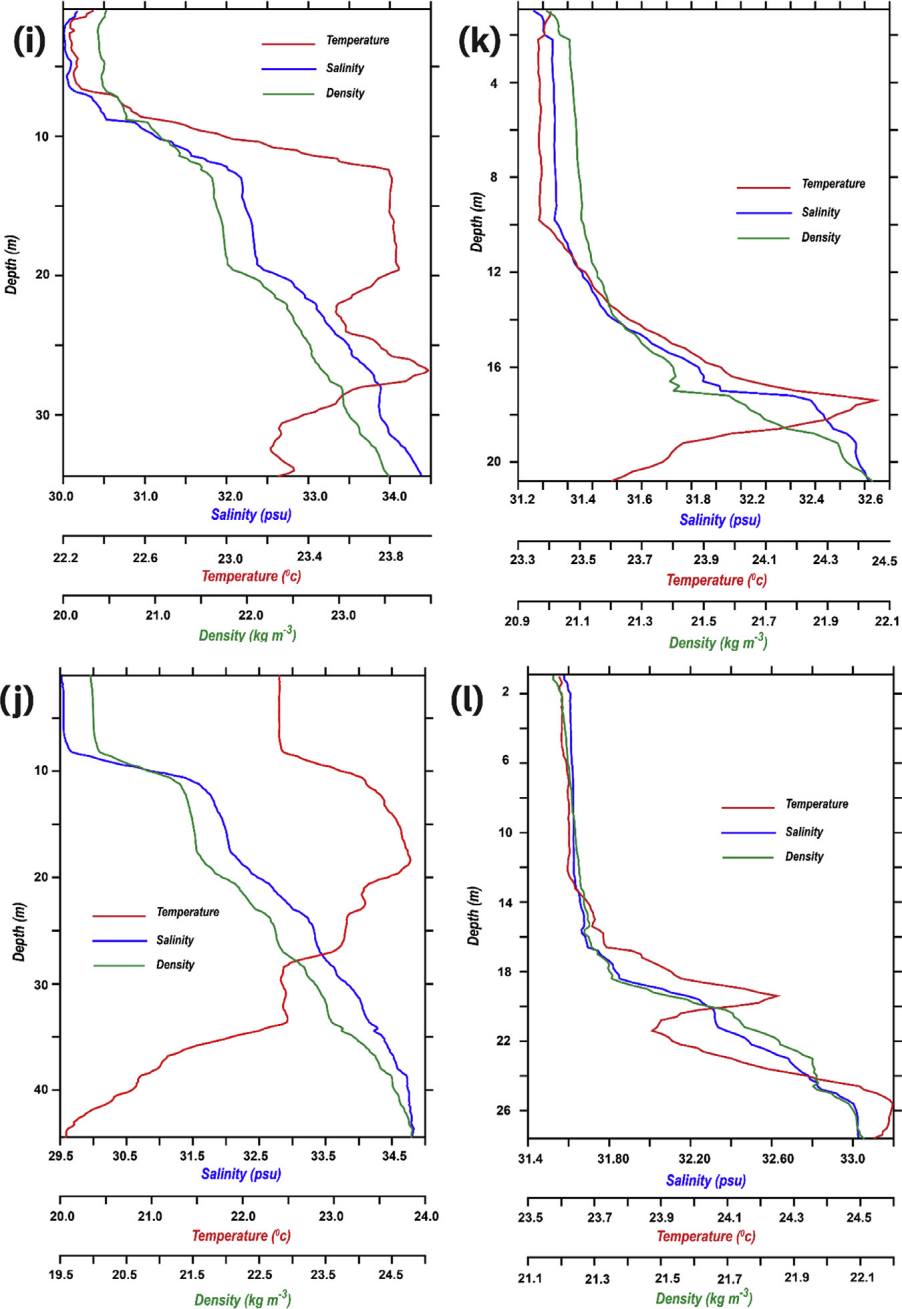
Combined vertical profiles of temperature (°C), salinity (psu), density (kg m^−3^) illustrated in red, blue and green lines respectively, of 12 stations: (a) Station 1, (b) Station 2, (c) Station 3, (d) Station 4, (e) Station 5, (f) Station 6, (g) Station 7, (h) Station 8, (i) Station 9, (j) Station 10, (k) Station 11, (l) Station 12.

Values staring from one meter was separately transferred to a text file, in order to transform it to a format that is generic to a wide variety of specific applications, ASCII (.dat) files. This kind of file usually stores information as either plain text or binary, hence making it easily modifiable with a text editor (e.g., Notepad++). These DAT files are read and further processed with Pyferret (7.5), an Ubuntu-based Python-Ferret module that provides Ferret's abilities to retrieve, manipulate, visualize, and save data. Ferret itself is interactive computer visualization and analysis environment designed and developed jointly by the National Oceanic and Atmospheric Administration (NOAA) and the Pacific Marine Environmental Laboratory (PMEL). Pyferret was later used to read ASCII (.dat) file of each station, it embedded and assigned four axes including latitude, longitude, depth, and time to each station's file (Fig. 3). Pressure values were considered to portray depth; therefore, each station's highest pressure was estimated to be the deepest point and finally converted to the depth axis in the program.

Once the axes were defined, the dataset was ready to be transformed into more of array-oriented data, network Common Data Form (netCDF) format. The entire transformation process is summarized in the schematic diagram (Fig.2). NetCDF (.nc) works as an interface and library at the same time [3], that provides creation, access, and sharing of array-oriented scientific data. Conversion to (.nc) format, made access to this data much easier as it is easily accessible by several programming languages including C, C++, Java, Fortran. Python, IDL, MATLAB, R, Ruby, and Perl also supports it with available programming interfaces [3].

### 2.3 Calculation of Mixed Layer Depth (MLD)

Generally, there are three methods of estimating MLD from ocean profile data; these are threshold methods, least squares regression methods and integral methods [4]. In correspondence to the threshold method, the depth at which the density (sigma-t) surpasses the surface value by 0.2 kg m^−3^, was considered as MLD [5] in this dataset. Pyferret was used to read netCDF (.nc) files of each station. In the program, density parameter was set as the threshold variable. Then an equation was set in a way that it finds 0.2 difference in the density array within each netCDF files and display the specific depth at which this change took place. That precise depth was identified as MLD (Table 2).

**Table 2:** Summary value of water column's physical properties derived from CTD profiles.

Serial no.	Date	Temperature (°C)		Salinity (PSU)		Density (kg m^−3^)		Mixed Layer Depth (m)
		*Mean*	*Standard Deviation*	*Mean*	*Standard Deviation*	*Mean*	*Standard Deviation*	
**1**	18/03/20	24.773	1.025	32.795	1.223	21.852	1.280	7.13333
**2**	8/02/20	23.613	0.859	32.131	1.074	21.632	0.596	7.93404
**3**	02/01/20	24.084	0.1097	32.230	0.385	21.558	0.295	4.62014
**4**	19/03/20	24.876	0.768	32.956	1.120	21.942	1.126	1.14347
**5**	19/03/20	25.108	0.796	32.706	1.07	21.672	1.094	6.45909
**6**	21/03/20	25.052	0.669	32.502	1.384	21.501	1.274	5.76597
**7**	9/02/20	22.167	1.286	32.961	2.059	22.728	1.801	7.77065
**8**	9/02/20	22.854	1.248	32.728	1.554	22.360	1.428	8.64324
**9**	9/02/20	23.285	0.591	32.275	1.444	21.864	1.015	8.23878
**10**	9/02/20	22.481	1.044	32.538	1.879	22.315	1.632	8.38739
**11**	9/02/20	23.602	0.297	31.671	0.455	21.287	0.303	13.4027
**12**	9/02/20	23.863	0.329	31.982	0.505	21.461	0.321	18.5828

## Declaration of Competing Interest

The authors declare that they have no known competing financial interests or personal relationships that could have appeared to influence the work reported in this paper.

## Appendix Supplementary materials

Image, application 1

Image, application 2
